# Efficacy and potential use of novel sustained release fillers as intracanal medicaments against *Enterococcus faecalis* biofilm in vitro

**DOI:** 10.1186/s12903-019-0879-1

**Published:** 2019-08-20

**Authors:** Bernhard Funk, David Kirmayer, Sharonit Sahar-Heft, Irith Gati, Michael Friedman, Doron Steinberg

**Affiliations:** 1 The Institute of Dental Sciences, Faculty of Dental Medicine, The Hebrew University of Jerusalem, Jerusalem, Israel; 2 The Institute of Drug Research, School of Pharmacy, Faculty of Medicine, The Hebrew University of Jerusalem, Jerusalem, Israel; 3 Department of Endodontics, Faculty of Dental Medicine, The Hebrew University of Jerusalem, Jerusalem, Israel; 4 Faculty of Dentistry, Hebrew University – Hadassah, PO Box 12272, 91120 Jerusalem, Israel

**Keywords:** Cetylpyridinium chloride, *E. faecalis* biofilm, Sustained release, Intracanal medication

## Abstract

**Background:**

*Enterococcus faecalis* is a bacterium frequently isolated after failed root canal therapy. This study analyzed the antibacterial and antibiofilm effects in vitro of sustained-release fillers (SRF) containing cetylpyridinium chloride (CPC) against vancomycin resistant *E. faecalis*.

**Methods:**

First, the solidification capability was tested by introducing liquid SRF into phosphate buffered saline, followed by 30 s of vortexing. The antimicrobial effects of SRF-CPC against static monospecies biofilms were analyzed with a metabolic assay. Inhibition of biofilm formation was tested by exposing daily refreshed *E. faecalis* suspensions to SRF-CPC for 9 weeks. To evaluate the effects of SRF-CPC against preformed biofilms, biofilms were grown for 1, 3 and 7 days, and then treated with SRF-CPC for 24 h. Biofilm kill time was tested by applying SRF-CPC to a 3-day-old biofilm and measuring its viability at different time points. All experiments were compared to Placebo SRFs and to untreated control biofilms. Data were analyzed with two-way ANOVA followed by Tukey’s test. Results were considered significant at *P* < 0.05.

**Results:**

The liquid SRF solidified within seconds and no structural changes were observed after 30 s of vortexing at maximum speed. SRF-CPC inhibited *E. faecalis* biofilm formation for 7 weeks and significantly reduced its viability in weeks 8 and 9. Mature biofilms grown for 1, 3 and 7 days were destructed by SRF-CPC in less than 24 h. Fifty percent of a 3-day-old biofilm was destructed in 2 h and complete destruction occurred in less than 12 h. (*P* < 0.05 in all cases, compared to SRII-Placebo).

**Conclusions:**

SRF-CPC’s physical properties and long-lasting anti-biofilm effects make it a promising coadjuvant medication for endodontic therapy.

**Electronic supplementary material:**

The online version of this article (10.1186/s12903-019-0879-1) contains supplementary material, which is available to authorized users.

## Background

*Enterococcus faecalis* is the most commonly recovered species from the root canals after failed root canal therapy (RCT) [1]. The primary goal of RCT is to remove all microorganisms from the inner surface of the root canal system, to prevent reinfection and to establish or maintain healthy periapical tissues [2]. Modern techniques and equipment have greatly contributed to increased clinical success rates and significantly shortened the time needed to complete the RCT, yet there are still limitations to the endodontic disinfection process; viable biofilm cells can persist in undertreated and untreated locations of the root canal system due to the inherent challenges associated with its complex anatomy [3–5]. Difficult clinical cases require more time, better skills of the practitioner and modern instruments for their treatment, and this can lead to the necessity of performing the RCT in more than one appointment [5]. Multiple patient visits might also be required to avoid the risk of flare-ups when periapical lesions are present [6]. Hence the use of intracanal medication is still a widely spread clinical practice to attain disinfection and prevent reinfection between appointments [7].

Ideally, an intracanal medicament should eliminate any remaining bacteria, reduce inflammation of periapical tissues, render canal contents inert and neutralize debris, act as a barrier against leakage from temporary filling, and help drying persistently wet canals [8]. Thus, the widely extended use of calcium hydroxide (CH) is reasonable [7, 9]. Still, despite its good physical, biological and pharmaceutical properties, CH cannot completely eradicate resilient intracanal microorganisms as *E. faecalis* [10]. One alternative to CH is chlorhexidine (CHX) in gel which was found to be very effective against *E. faecalis* [11]*.* However, CHX gel does not act as a physical barrier when injected into the root canal, nor does it inactivate lipopolysaccharides (LPS), a wall component and degradation product of gram-negative cells, which, when persisting, causes periapical inflammation, bone resorption and pain [12–14].

Another antibacterial agent, only recently tested for endodontic purposes, is cetylpyridinium chloride (CPC), a cationic quaternary compound, commonly found in mouthwashes [15]. Incorporated to dental cements and gutta-percha points or used as irrigation solution, it proved to be very effective against *E. faecalis* [16–18]. Furthermore, a recent study showed that repeated exposure of *E. faecalis* to CHX led to its resistance, whereas CPC did not elicit such deleterious response [19]. Another important feature that favors CPC over CHX for endodontic applications is its inhibiting effect on LPS binding to the toll-like receptor 4 involved in inflammatory cytokine production [20].

Nevertheless, good antibacterial effects of a drug alone are not sufficient against endodontic biofilms. The penetration of a drug into deeper layers of the biofilm is limited by extracellular polysaccharides (EPS) that act as an affinity matrix, increase viscosity of the medium, and thus slow down the diffusion of antimicrobials [21]. Nevertheless, higher concentrations of antimicrobial agents at the outer layer of the biofilm should increase the overall diffusion rates into the deeper layers [22]. To achieve high local concentrations over an extended period of time, a drug can be incorporated into a locally-administrable type of formulation or device that controls its local release at the site of action [23, 24]. Numerous sustained release devices have already been developed, tested and commercialized in different fields of dentistry [22]. This concept of controlled release can also be applied to the root canal system [25].

Phaechamud et al. [26] have recently described a solvent exchange-induced in situ forming gel, based on pharmaceutical ammoniomethacrylate type b, for periodontitis treatment. However, to be applicable to RCT, the formulation should remain in the area for the period sufficient to eradicate the infection, have sufficient penetration power, yet seal off the canal and fill it completely. Hence, in this study we tested a novel concept of sustained-release fillers (SRFs) containing CPC (SRF-CPC), based on the polymers used by Phaechamud et al. [26]. Our approach encompasses the use of pharmaceutical polymers dissolved in acceptable solvents thus providing injectability into the root canal. Due to carefully chosen additives, unlike in-situ gels, our SRF solidifies very rapidly upon contact with aqueous medium. Our main goal for the current study was to analyze the sustained antibacterial effects of SRF-CPC against *E. faecalis* biofilm in vitro. The null hypothesis of the study was that a controlled-release filler comprising CPC cannot effectively eradicate biofilms or inhibit growth of microbiota implied in root canal infections for extended time intervals.

## Methods

### Preparation of the SRF

The detailed preparation procedure is set forth in the Additional file 1. Briefly, the heat-sterilized ammoniomethacrylate copolymer, type A, according to the United States Pharmacopeia 41 – National Formulary 36, was dissolved in sterile-filtered N-methyl pyrrolidone - water mixture with CPC and a small amount of calcium chloride. The resulting formulation contained 0.5% of CPC. The formula of the composition is summarized in Table 1. The pharmaceutical release profile of CPC for the SRF can be found in Additional file 2.

**Table 1:** Composition of the tested SRF-CPC (10-g formulation)

Components	SRF-CPC (mg)
CPC	50
Ammoniomethacrylate copolymer	2000
Calcium chloride	200
DDW	400
NMP	7350

### Solidification of SRFs

Solidification of the SRF was performed by injecting about 200 μL of the composition into a scintillation vial containing about 10 mL of phosphate buffer according to the USP (i.e. as defined in the United States Pharmacopeia), at pH 6.8. The immediately solidified filler was vortexed for about 30 s at maximal velocity, to observe minimal or no shape change. The solidified residue was soft spongiform solid, maintaining the original shape upon flow but yielding to mechanical tools.

### Handling of SRFs

**Fig. 1:**
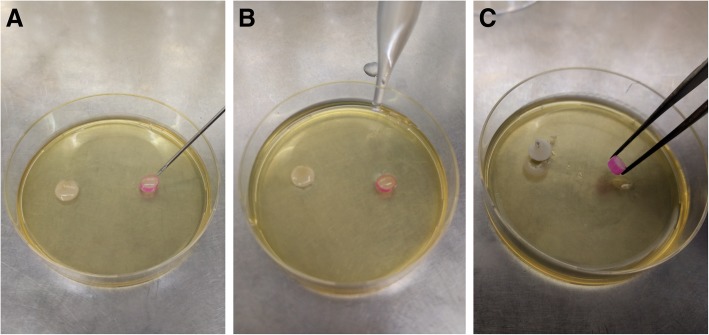
Preparation of SRF specimens for in vitro use. **a** SRF is injected as a gel into a segmented tube reservoir placed on an agar plate. **b** PBS is added until completely covering the specimen. **c** After 5 min the solidified SRF specimen can be easily transferred with sterile tweezers for use in experiments

SRFs were solidified on agar plates to allow consistent shape and ease of handling during experimentation, as follows: polypropylene tubes of 6 mm diameter were segmented into 2-mm pieces, sterilized and placed on small 60-mm petri dishes (Miniplast, Ein Shemer, Israel) containing 9 mL brain heart infusion agar (BHI agar; Neogen Corporation, Lansing, Michigan, USA). Aliquots of 30 μL of SRF-CPC (or SRF-Placebo if needed) were then separately dispensed into their respective reservoirs using syringes and 23-Gauge needles. Contact of the SRF with the agar initiated solidification. To complete solidification, 9 mL of sterile phosphate buffered saline (PBS; Sigma Life Sciences, St. Louis, MO, USA) were carefully added to the agar plate to assure full immersion of the SRFs. After 5 min SRFs were removed and used immediately for the experiments. The steps can be seen in Fig. 1.

### Antibiofilm effects of SRF-CPC

#### Biofilm growth

To grow *E. faecalis* biofilms we followed the initial steps of a protocol presented by Kwasny and Opperman [27] for the growth of static biofilm of non-motile gram-positive bacteria, with some minor modifications. The method of biofilm growth described here, was used for all following experiments in the current study. Briefly, cultures of *E. faecalis* V583 grown in an orbital shaker overnight in aerobic conditions at 37 °C were diluted 1:50 (OD_600_ ~ 0.1) with fresh BHI broth containing 2% glucose [28]. Aliquots of 270 μL of this bacterial suspension were used for biofilm formation inside the individual wells of a sterile polystyrene 48-well tissue culture plate (TCP; SPL Life Sciences, Pocheon-si, Gyeonggi-do, Korea). TCPs were then incubated at 37 °C in aerobic conditions for 24 h.

#### Inhibition of biofilm formation

To evaluate the long-term antibacterial effects of SRFs against biofilm formation de novo, daily renewed bacterial suspensions intended for biofilm growth were exposed to specimens of either SRF-CPC or SRF-Placebo for 9 weeks. SRF specimens were prepared as described in the section above “Handling of SRFs” and placed into the individual wells of a 48-well TCP containing 270 μL of bacterial suspension. Each day during the test period, the supernatant-fluid surrounding the SRFs was removed and replaced by 270 μL of fresh bacterial suspension for biofilm growth. Specimens of SRF-CPC and SRF-Placebo were transferred to the wells of a new TCP under sterile conditions every 7 days. The grown biofilms located in the wells of the used plate were then analyzed with the MTT assay (3-(4,5-dimethylthiazol-2-yl)-2,5-diphenyltetrazolium bromide reduction assay) described in a section below. Control biofilms in this experiment were grown without addition of SRFs.

#### Biofilm destruction

This experiment was performed to assess the ability of SRF-CPC to destroy pre-grown biofilms. Biofilms were grown in separate TCPs for either 1, 3 or 7 days. Media, 270 μL of BHI with 2% glucose, was replaced every day. The pre-grown biofilms inside the individual wells were then exposed to either one specimen of SRF-CPC or SRF-Placebo for 24 h. Next, the biofilms were analyzed with an MTT assay to measure the antibiofilm effects of the SRFs. Control biofilms were grown without addition of SRFs.

#### Biofilm kill time

To determine the time necessary to destroy a mature existing biofilm, pre-grown mature biofilms were exposed to SRFs and evaluated at different time points. The experiment was performed as follows. Biofilms were grown for 3 days and media replaced every 24 h with 270 μL of fresh BHI broth supplemented with 2% glucose. Mature biofilms were then exposed to specimens of SRF-CPC or SRF-Placebo for either 1/2, 1, 2, 6 or 12 h. Next, an MTT assay was performed. Control biofilms were not exposed to SRFs.

#### MTT assay

This method allows to measure the metabolic activity of viable cells that compose the biofilm. The protocol employed by Walencka et al. [29] was slightly modified as follows. In brief, at the end of each experiment, after microscopically confirming the absence of contaminating microorganisms, SRFs were removed with sterile tweezers and the wells were washed twice with 200 μL of PBS solution to remove planktonic cells. Aliquots of 50 μL of 0.1% w/v MTT (Calbiochem, Darmstadt, Germany) were then added to each well to cover the biofilm. After 1 h of incubation at 37 °C in aerobic conditions, the excess of MTT was washed with 150 μL of PBS. Next, 200 μL of dimethyl sulfoxide (DMSO; Bio-Lab, Hayetzira, Jerusalem, Israel) were added to each well and the plate was placed on an orbital shaker (S-3.02.10 M, ELMI, Riga, Latvia) for 10 min at room temperature. Aliquots of 150 μL of the solubilized MTT solution were transferred to unused wells of a 96-well plate (SPL Life Sciences, Pocheon-si, Gyeonggi-do, Korea). Great care was taken to avoid aspiration of polymer debris and biofilm masses on the bottom of the wells. Finally, absorbance was measured at 540 nm with a reference wavelength of 630 nm using an Infinite 200 PRO spectrophotometer (Tecan Austria, Grödig, Austria).

### Statistical analysis

Statistical analysis was performed using GraphPad Prism version 7.04 (GraphPad Software, La Jolla, CA, USA). Results from the biofilm experiments were analyzed with a two-way ANOVA test to detect overall statistical differences between test groups, followed by Tukey’s test to identify specific differing groups. All data were expressed as mean with standard deviation and considered significant at *P* < 0.05. Supporting data were provided in Additional file 3.

## Results

### Solidification of SRF-CPC ensues quickly

**Fig. 2:**
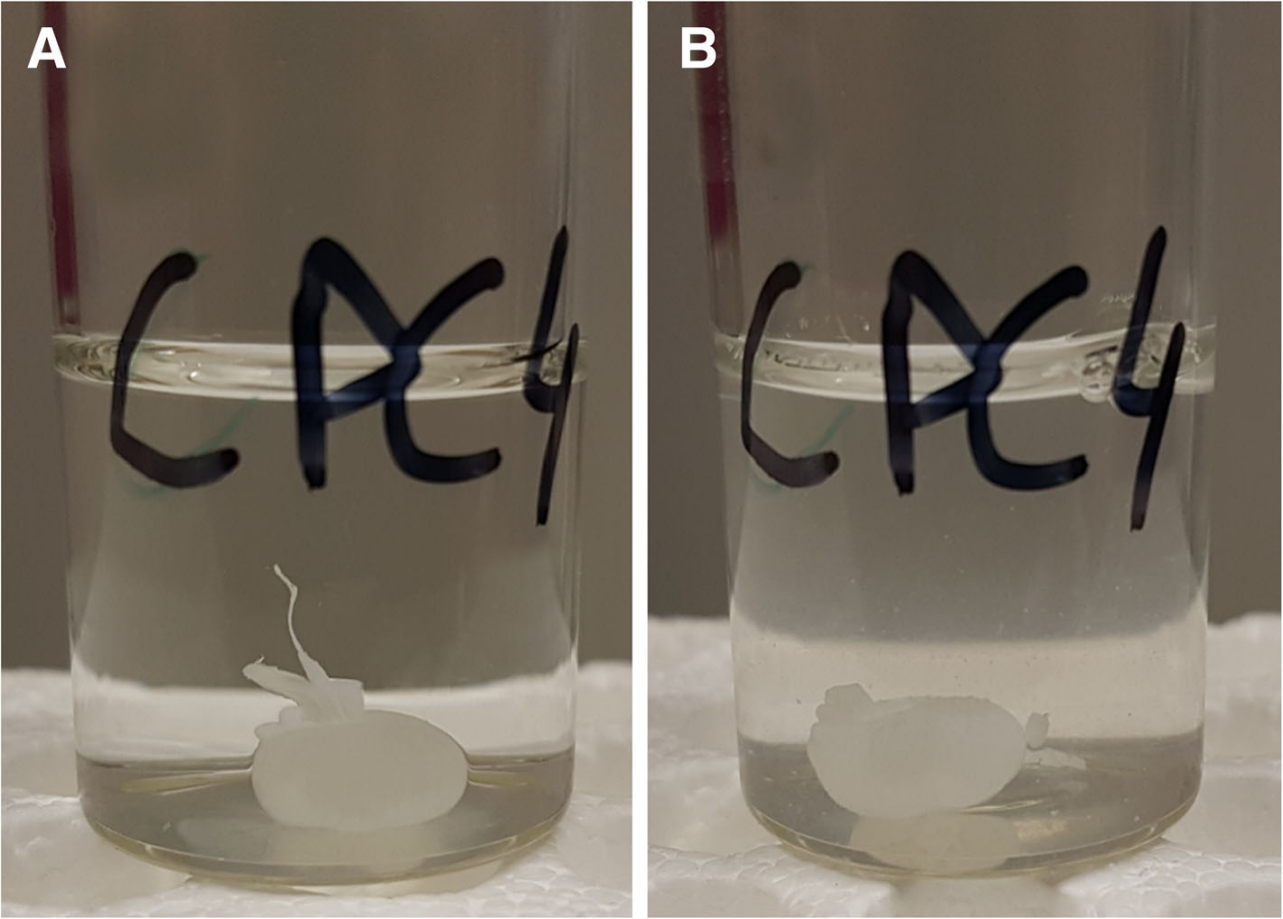
Solidification of SRF-CPC. **a** About 20 s after injection. **b** After another 30 s of vigorous mixing

The formulation solidified within several seconds upon contact with phosphate buffer USP, pH 6.8. Mixing thoroughly for 30 s resulted in no structural change of the of the solidified medicament. The photographs are shown in Fig. 2.

### SRF-CPC inhibits biofilm formation for 7 weeks in multi-well plates

**Fig. 3:**
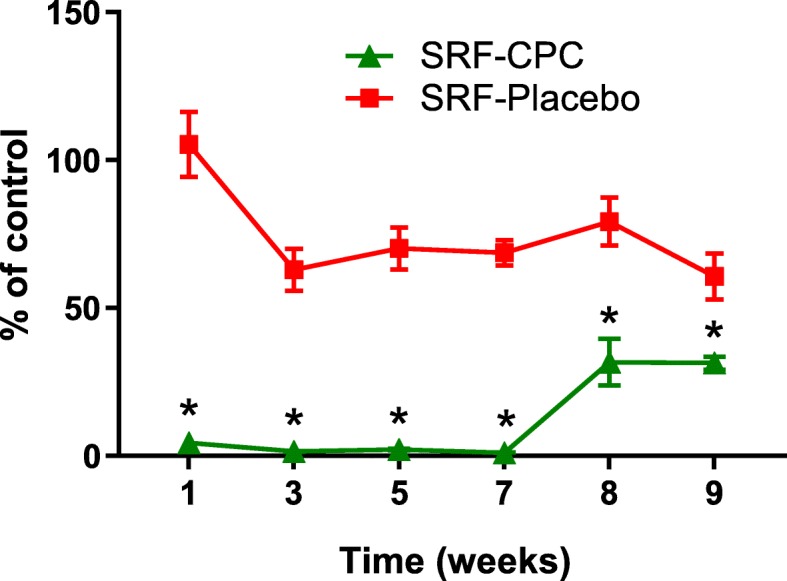
Percentages of biofilm viability in presence of SRF-CPC and SRF-Placebo. Data expressed as mean with standard deviation. *P < 0.05, compared with SRF-Placebo and control sample; *n* = 6

To assess the long-term effects of SRF-CPC and SRF-Placebo on biofilm formation, metabolic activity was measured once a week with the MTT assay. SRF-CPC inhibited biofilm formation for 7 weeks and during the following 2 weeks a significant reduction of biofilm activity was observed when compared to the placebo and control wells (Fig. 3). SRF-Placebo produced a significant decrease in biofilm formation on the bottom surface of the microwells compared to the control sample after the first week.

### SRF-CPC terminates metabolic activity of biofilms at different maturity stages

**Fig. 4:**
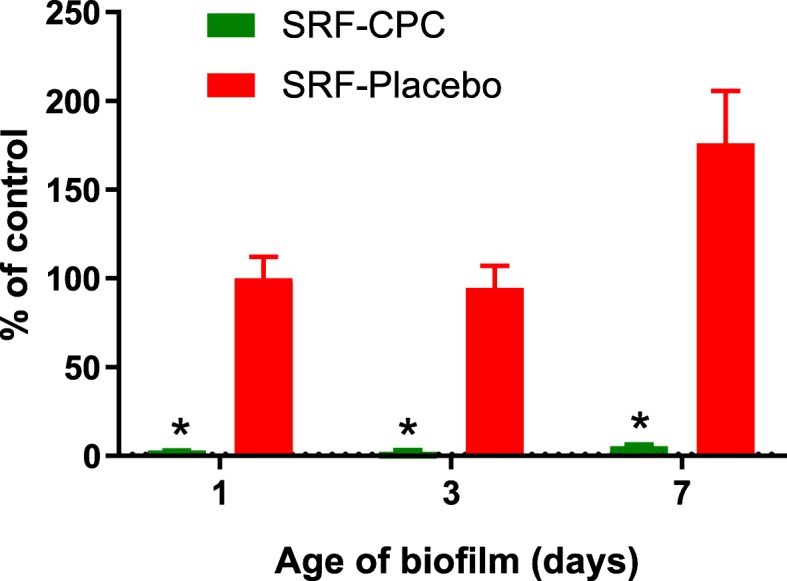
Percentage of pre-grown biofilm viability after exposure to SRF-CPC and SRF-Placebo during 24 h. Data expressed as mean with standard deviation. *P < 0.05, compared with SRF-Placebo and control sample; *n* = 4

SRF-CPC was able to destruct biofilms that where pre-grown for either 1, 3 or 7 days, regardless of their maturity stage, whereas SRF-Placebo alone did not significantly reduce biofilm viability at any stage (Fig. 4).

### SRF-CPC terminates metabolic activity of mature biofilms in less than 12 h

**Fig. 5:**
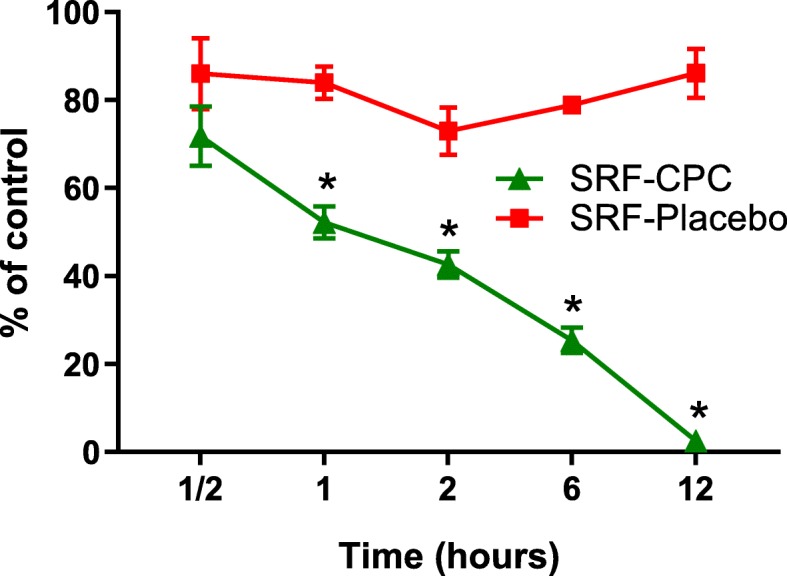
Percentage of mature biofilm viability after exposure to SRF-CPC and SRF-Placebo at increasing time periods. Data expressed as mean with standard deviation. *P < 0.05, compared with SRF-Placebo and control sample; *n* = 4

The results displayed in Fig. 5 show a clear decrease in viability of the biofilm as time progresses. Around 50% of the biofilm was destructed by SRF-CPC after only 2 h and no metabolic activity was detected after 12 h. SRF-Placebo failed to destruct *E. faecalis* biofilm viability, however a significant reduction in metabolic activity was observed after 1 h onwards in comparison to the control sample.

## Discussion

The present results indicate that the null hypothesis must be rejected.

Many improvements have been made lately in the chemico-mechanical preparation of the root canal. Rotary files with optimized taper sizes remove more infected dentin and make it possible to prepare the root canals that favor effective irrigation [30, 31]. The removal of biofilm from the inner walls of the canal is further enhanced when irrigation is assisted by ultrasonic or laser activation [32, 33]. However, several clinical studies have shown no significant clinical advantage of these procedures over conventional protocols [34–36] and the extent in which they affect biofilms located in remote regions of oval-shaped canals, lateral canals and tubules, is still unknown [37]. Hence, intracanal medicaments are inserted into root canals intending to restrict bacterial regrowth and supply continued disinfection [38]. Ideally such medicaments should deliver and maintain significant concentrations of antimicrobial agent during the time period between RCT sessions; this is the role that the sustained-release formulations can readily play.

Due to the versatility of pharmacopeial methacrylate polymers it is possible to design sustained release formulations with tailor-made release profiles, to which a drug of choice can be added, in our case, CPC. The efficacy of CPC against dental plaque as component of sustained release films was already confirmed in a clinical study published by Friedman et al. [39]; here, for the first time we tested CPC as component of a novel sustained release solidifying filler intended for endodontic use. Using the currently reported approach, we have successfully reached drug release at controllable rate of CPC. The initial drug concentration of 0.5% is in line with the present practice; however when the SRF solidifies and the solvent leaches out from SRF and partially switches for water, this initial concentration in the solution becomes higher in the solid SRF. Nevertheless, due to the controlled release properties of the SRF, particularly in restrictive conditions where the extraction solvent is not readily available, the amounts of active agent released in that solvent are relatively low, allowing for the pronounced inhibition period demonstrated in the present work.

As with any antiseptic, also the use of CPC may raise a question of possible cytotoxicity. The general outnumbering principle of antiseptic use, which stipulates that when killing one tissue cell, the tissue has one cell less, but when killing one bacterium cell, the bacterium is dead, generally proves true, which lead the regulators already over two decades ago to believe that antiseptics, inter alia CPC, are safe for use on oral mucosa [40]. Yet, the cytotoxicity of CPC cannot be completely ignored. Depending on the tested cell type, the half-population cytotoxic concentration of CPC (CC_50_) was found to vary between 0.003 and 0.001% [41, 42]. On the other hand, the minimum inhibitory concentration (MIC) and the minimal biocidal concentration (MBC) of CPC for *E. faecalis* in our experimental conditions was estimated as 0.0003 and 0.0006%, respectively (data not shown), whereas Tomino et al. [18] reported the MIC value of 0.0001% with CPC incorporated into agar plates. This means that there is a window of effective concentrations between 3 × 10^− 4^% and 10^− 3^% before any cytotoxic effect could even potentially be seen. Due to flexibility of the pharmaceutical formulation, the concentration of CPC in practice could be readily adjusted to any desire value. Despite the total concentration of CPC in SRF-CPC being 0.5%, it is impossible to know at this stage what concentrations of CPC could be obtained in the root canal throughout the drug release period, which we have seen can be as long as 7 weeks at effective antibacterial concentrations; the dose spread over such time interval effectively brings the amounts of CPC released to the safe range. At the minute amounts of solvents that would be available in the root canal, it is believed that there will be even less release of CPC.

The cationic nature of the polymer used in the formulation may play a certain role in its antimicrobial effect. It was demonstrated that ammoniomethacrylates polymers USP, are capable of promoting cell growth on surface, in particular the adhesion, proliferation and differentiation of human mesenchymal stem cells [43]. We have also seen that certain other types of adhering cells attach to the polymer, regardless of the physical form, i.e. either as a film or on supported particles. Therefore, we believe that the viable bacteria may be attracted to the constitutive positive charge on the surface of the SRF-CPC, and thus be exposed to higher concentration of CPC – at the surface of the SRF. This is supported by the fact that placebo filler decreased the amount of biofilm grown on the TCP well, which can only be explained that the surface of the filler attracts the viable bacteria, until it is completely covered with biofilm. This decoy mechanism may explain the long duration efficacy of the formulation against *E. faecalis*, but comparative studies with other polymers may be needed to prove unequivocally that the surface charge may attract bacteria to these drug delivery systems.

The long-lasting antibacterial efficacy of SRF-CPC is fundamental for clinical applicability as the time between endodontic appointments can range from a few days to several weeks, since maintaining a clean and disinfected root canal after initial treatment until the next visit is one of the main concerns in endodontic therapy. Our results show that single doses of SRF-CPC were able to inhibit *E. faecalis* growth and biofilm formation for 7 weeks in vitro, in a biological set-up where media was changed every day and fresh bacterial cells were added. In complex root canals, biofilms can persist in areas that escape instrumentation and irrigation. Our findings suggest that any surviving *E. faecalis* biofilm left inside the root canal system would be exposed to high local concentrations of CPC during the first days. SRF-CPC destructed 50% of mature 3-day-old *E. faecalis* biofilms in only 2 h and 7-day old biofilms in less than 24 h. During the following months CPC concentrations would be reinforced and maintained by a continuous drug release from the SRF-CPC. An initial burst effect followed by a more graduate release rate of CPC would drastically decrease the probability of *E. faecalis* biofilm survival.

Besides its significant antimicrobial/antibiofilm effects, SRF-CPC also presents good physical properties. Its viscosity allows it to be easily ejected through a 23-Gauge needle. Humidity does not constitute an obstacle and only contributes to SRF-CPC’s solidification. In a clinical setting, humidity present on the internal walls of the root canals and in the apical end of the root is expected to quickly start the solidification process of SRF-CPC and encapsulate the remaining solution, hence preventing unwanted leak to the periapex. In addition, it is expected to block penetration of periapical fluids into the root canal and act as a physical and pharmaceutical barrier against leakage coming from a poor temporary obturation at the coronal end. SRF-CPC’s soft consistency should facilitate its effortless removal using endodontic files and irrigation/aspiration. Nevertheless, more testing must be done to confirm SRF-CPC’s applicability for RCT in clinical settings.

The concept of using sustained release drugs as an adjunctive to the intracanal procedure has been reported in the past using different pharmaceutical technologies than proposed in this study. Heling et al. [44] successfully tested a sustained release device in a form of a strip containing chlorhexidine, with the capability to swell in the presence of liquids. Their device prevented *E. faecalis* biofilm formation ex vivo, even after two reinfections during a 7-day period. Huang et al. [45] tested a cylindrical, needle-shaped device prepared with ethyl cellulose and loaded with CHX. They reported a relatively steady release for over 40 days, similar to the results of our study. After 7 days of incubation with the device placed inside the canal of a previously infected bovine tooth, no microbial growth of *E. faecalis* was detected. One disadvantage of prefabricated devices is the potential need for modifications in shape and length to fit into particular root canals. The developed injectable filler SRF-CPC easily adopts the shape of its containing cavity. More recent studies focused on the use of micro- and nanoparticles. Polymeric biodegradable microspheres of poly (lactic-co-glycolic-acid) (PLGA) and zein loaded with amoxicillin were able to release the drug over 6 days at significant levels [46]. Mesoporous calcium-silicate nanoparticles loaded with CHX had excellent antibacterial and in vitro mineralization properties [47]. PLGA-moxifloxacin nanoparticles were also shown to be effective against *E. faecalis* for at least 14 days [48]. However, concerns about the potential toxicity of nanoparticles have been voiced in the literature, as the interactions at the nano-bio interface may impact the function of biomolecules, cellular components and tissue structures, well beyond the contemplated drug delivery paradigm [49]. Finally, a study performed by Gandhi et al. [50] presented a sol-gel device, consisting of GELRITE Gellan polymer loaded with CHX. This device would release the drug only in an acidic inflammatory environment, hence preventing premature peaking of the drug associated with possible side effects. Whether such a feature would be of clinical relevance or even desirable inside the root canals is yet to be unequivocally determined.

The quest for sustained release alternatives to common intracanal medicaments has been ongoing for nearly 30 years. Despite its excellent antibacterial and antibiofilm effects, especially against vancomycin-resistant *E. faecalis*, the consideration of CPC as constituent of sustained release drugs in endodontics remained mostly unexplored. The well-established use of calcium hydroxide may be appropriate for most clinical cases, however, when confronted with refractory periapical lesions due to the persistence of *E. faecalis*, a different intracanal medicament should be selected. The SRF-CPC presented in this study has promising antimicrobial, pharmacological and physical properties, essential against endodontic *E. faecalis* biofilms.

## Conclusions

Due to its good physical properties, its efficacy against mature biofilms and its long-lasting antibacterial effects against vancomycin resistant *E. faecalis*, SRF-CPC has the potential to become a significant coadjuvant medication to root canal therapy.

## Additional files

Additional file 1: Preparation and drug release of SRF-CPC. The file contains a detailed preparation procedure of the SRF, and the release of CPC measurements. (DOCX 13 kb)

Additional file 2: Release profile of SRF-CPC. The file contains a figure with the results of the release profile of CPC from SRF-CPC. (TIF 381 kb)

Additional file 3: Supporting data. The file contains data to support Figs. 3, 4 and 5. (XLSX 30 kb)

## Data Availability

The datasets supporting the conclusions of this articles are included within the article and its additional files.

## Abbreviations

BHI: Brain heart infusion
CH: Calcium Hydroxide
CHX: Chlorhexidine
CPC: Cetylpyridinium chloride
EPS: Extracellular polysaccharides
LPS: Lipopolysaccharide
MBC: Minimum bactericidal concentration
MIC: Minimum inhibitory concentration
MTT: 3-(4,5-dimethylthiazol-2-yl)-2,5-diphenyltetrazolium bromide reduction assay
PBS: Phosphate buffered saline
RCT: Root Canal Therapy
SRF: Sustained release filler
SRF-CPC: Sustained release filler containing cetylpyridinium chloride
SRF-Placebo: Placebo sustained release filler
TCP: Tissue culture plate
USP: United States Pharmacopeia
ZOI: Zone of inhibition
