# Supplementary material for: Efficacy and potential use of novel sustained release fillers as intracanal medicaments against Enterococcus faecalis biofilm in vitro
Source: BMC Oral Health. 2019 Aug 20;19:190. doi: 10.1186/s12903-019-0879-1 (PMC6700812; doi:10.1186/s12903-019-0879-1)
Supplement: Supplementary file 1 — Preparation and drug release of SRF-CPC. The file contains a detailed preparation procedure of the SRF, and the release of CPC measurements. (DOCX 13 kb) [file 12903_2019_879_MOESM1_ESM.docx]

**Preparation of SRF-CPC**

We obtained Eudragit polymers as free samples from Israeli representatives of Evonik® (Wilhelm Rosenstein, Herzlia, Israel). Specifically, Eudragit L and RL were used, corresponding to polymethacrylates type A, USP (i.e. as defined in the United States Pharmacopeia), and ammoniomethacrylates type A, USP. Calcium chloride was obtained from Merck, Israel; cetylpyridinium chloride (CPC), and N-methyl pyrrolidone (NMP) were obtained from Sigma-Aldrich, Israel; water was used purified from ion-exchange column and further redistilled.

The SRGs were prepared as follows: calcium chloride was dissolved in minimum amount of water and diluted with about 80% of the required volume of N-Methyl-2-pyrrolidone (NMP). Next polymers were added while vigorously mixing with a Vortex mixer and placed for about 15 minutes into an oven heated to 40°C, until complete dissolution of the polymers. Thereafter, CPC was added to the solution using Vortex mixer, and the remainder of the solvent was added to facilitate dissolution. The solution was left standing at 40°C for another 15 minutes to ensure complete dissolution and was stored at room temperature until used. For the aseptic preparation, the weighed amount of polymers was dissolved in ethanol and cast onto Petri dishes in an aseptic environment; the solution of CPC and calcium chloride in NMP with minimum amount of water was sterile-filtered using a 0.22 μm Nylon filter into a sterile vial containing the dried polymer aseptically cut into small pieces. The mixture was vigorously mixed and placed in an oven at 40°C for about 30 minutes to complete dissolution.

**CPC release from the SRF**

To assess the kinetics of the CPC release from the SRF, about 100 μL of the solution were injected into 2.5 mL of phosphate buffer according to the United States Pharmacopeia (USP), at pH 6.8, preheated to 37 °C. The release medium was completely changed between the samples to maintain sink conditions, at 15, 30, and 60 minutes, and then hourly until 8 hours, followed by 24 hours. The CPC concentrations were analyzed by HPLC.

The release profile is demonstrated in the figure submitted as additional file titled “Additional file.tif”. It can be seen that even at sink conditions (when there is enough solvent to extract 10 times the amount of drug in the dosage form) the release kinetics is moderate, following initial burst release, allowing delivery of CPC for prolonged time intervals.
