# Supplementary figures and images for: Efficacy and potential use of novel sustained release fillers as intracanal medicaments against Enterococcus faecalis biofilm in vitro
Source: BMC Oral Health. 2019 Aug 20;19:190. doi: 10.1186/s12903-019-0879-1 (PMC6700812; doi:10.1186/s12903-019-0879-1)

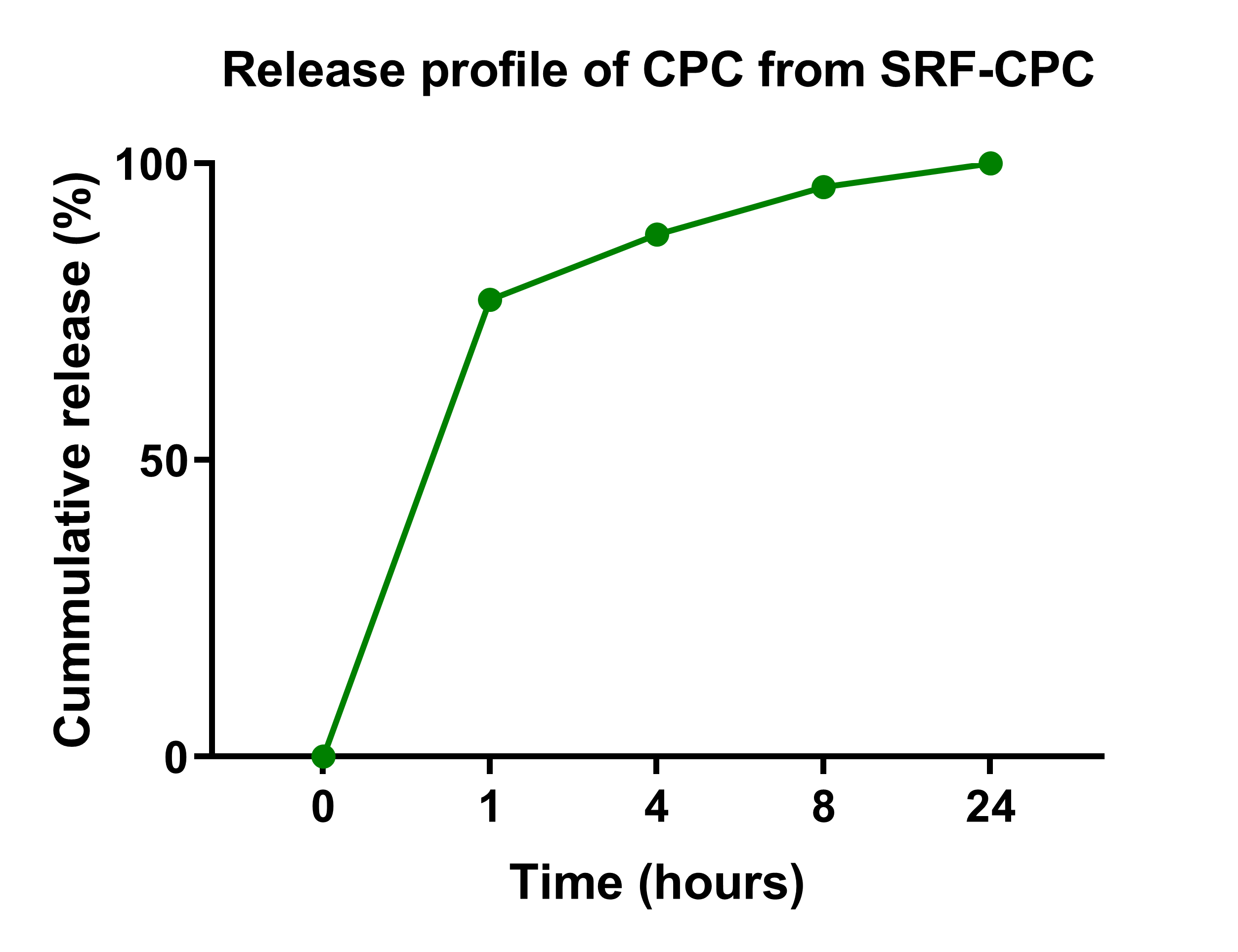

Supplement: Supplementary file 2 — Release profile of SRF-CPC. The file contains a figure with the results of the release profile of CPC from SRF-CPC. (TIF 381 kb) [file 12903_2019_879_MOESM2_ESM.tif]
